# What value is the CINAHL database when searching for systematic reviews of qualitative studies?

**DOI:** 10.1186/s13643-015-0069-4

**Published:** 2015-06-26

**Authors:** Kath Wright, Su Golder, Kate Lewis-Light

**Affiliations:** 1 Centre for Reviews & Dissemination, University of York, York, UK; 2 Department of Health Sciences, University of York, York, UK

## Abstract

**Background:**

The Cumulative Index to Nursing and Allied Health Literature (CINAHL) is generally thought to be a good source to search when conducting a review of qualitative evidence. Case studies have suggested that using CINAHL could be essential for reviews of qualitative studies covering topics in the nursing field, but it is unclear whether this can be extended more generally to reviews of qualitative studies in other topic areas.

**Methods:**

We carried out a retrospective analysis of a sample of systematic reviews of qualitative studies to investigate CINAHL’s potential contribution to identifying the evidence. In particular, we planned to identify the percentage of included studies available in CINAHL and the percentage of the included studies unique to the CINAHL database. After screening 58 qualitative systematic reviews identified from the Database of Abstracts of Reviews of Effects (DARE), we created a sample set of 43 reviews covering a range of topics including patient experience of both illnesses and interventions.

**Results:**

For all 43 reviews (21 %) in our sample, we found that some of the included studies were available in CINAHL. For nine of these reviews, all the studies that had been included in the final synthesis were available in the CINAHL database, so it could have been possible to identify all the included studies using just this one database, while for an additional 21 reviews (49 %), 80 % or more of the included studies were available in CINAHL. Consequently, for a total of 30 reviews, or 70 % of our sample, 80 % or more of the studies could be identified using CINAHL alone. 11 reviews, where we were able to recheck all the databases used by the original review authors, had included a study that was uniquely identified from the CINAHL database. The median % of unique studies was 9.09 %; while the range had a lowest value of 5.0 % to the highest value of 33.0 %.

**Conclusions:**

Assuming a rigorous search strategy was used and the records sought were accurately indexed, we could expect CINAHL to be a good source of primary studies for qualitative evidence syntheses. While we found some indication that CINAHL had the potential to provide unique studies for systematic reviews, we could only fully test this on a limited number of reviews, so we are less confident about this finding.

**Electronic supplementary material:**

The online version of this article (doi:10.1186/s13643-015-0069-4) contains supplementary material, which is available to authorized users.

## Background

The selection of databases used when carrying out literature searching for a systematic review can have a significant impact upon the number of records retrieved and the subsequent stages of the review in terms of time and resources spent as well as the review’s conclusions. While wishing to identify all the available relevant studies to minimise potential bias, researchers and information specialists also want to minimise the number of irrelevant records retrieved so the choice of databases is a key decision.

Descriptions of database content are included in several guides to conducting systematic reviews [1–3], but there is no guidance on the optimum choice of databases. Indeed, as reviews are now carried out in a wide range of specialised topic areas such as diagnostics and prognostics, public health, adverse effects and economic evaluations, it would be difficult to make recommendations that would be appropriate in all circumstances.

Database descriptions provided by database producers may list the subject areas covered but tend to provide less information about coverage in terms of methodological types. Examining available publication-type tags and thesaurus terms can provide more information about database coverage and help in the choice of databases to search.

Shaw’s [4] comparison of thesaurus terms for qualitative research across MEDLINE, Embase, Cumulative Index to Nursing and Allied Health Literature (CINAHL), British Nursing Index, Applied Social Sciences Index and Abstracts (ASSIA) and Social Science Citation Index shows that in 2004 both CINAHL and MEDLINE included the same (or broadly similar) thesaurus terms for *qualitative research* (MEDLINE), *qualitative studies* (CINAHL), *questionnaires* (MEDLINE & CINAHL), *focus groups* (MEDLINE & CINAHL) and *attitude* (MEDLINE & CINAHL). However, CINAHL also included the following thesaurus terms not available in MeSH: *discourse analysis, content analysis, ethnographic research, ethnological research, ethnonursing research, constant comparative method, qualitative validity, purposive sample, observational methods, field studies, theoretical sample, phenomenology, phenomenological research, life experiences* and *cluster sample*. When we re-checked these thesaurus terms in 2014 against both the Medical Subject Headings (MeSH) and the CINAHL thesaurus, we found that there was no substantial change i.e., there were a significant group of terms describing qualitative research that were available in CINAHL but not in MEDLINE.

In addition, in examining the subheadings of the CINAHL thesaurus term *“qualitative studies*” (*action research, ethnographic research, ethnological research, ethnonursing research, grounded theory, naturalistic inquiry, phenomenological research*) and checking which of these was available in the MeSH thesaurus, we found that only “*grounded theory*” was included in MeSH.

It is considered that some databases are more useful than others for specific types of review, and CINAHL is generally thought to be a good source to search when conducting a review of qualitative evidence [5–7]. Its thesaurus includes a range of terms relating to qualitative research methods e.g., qualitative studies, action research, naturalistic enquiry and ethnographic research suggesting that its coverage of qualitative research could be more extensive than other databases.

This could suggest that, when seeking to identify qualitative research studies, CINAHL may be a better choice of database than MEDLINE.

Two case studies have looked at CINAHL’s performance. One study by Subirana et al. 2005 [8] found that it was essential to search both CINAHL and MEDLINE to identify all relevant included studies in a systematic review covering a nursing topic. Another paper by Flemming et al. 2007 [5] tested the recall when searching in a review of patients’ experiences of living with a leg ulcer. In this case study, seven bibliographic databases (MEDLINE, CINAHL, Embase, British Nursing Index (BNI), Social Science Citation Index (SSCI), ASSIA and PsycINFO) were searched using three different strategies—one consisting of thesaurus terms, the second composed of free-text terms and the third using broad-based terms. The authors concluded that the simple search strategy (i.e., the broad based, consisting of just three search terms) was just as effective as either of the more complex ones. In addition they found that “It may be feasible to restrict searches with a clear nursing focus to the CINAHL bibliographic database” as it was the only one of the seven databases to identify all of the included studies using any one of the three search strategies being tested.

While these two examples suggest that using CINAHL could be essential for reviews of qualitative studies covering topics in the nursing field, it is unclear whether this can be extended more generally to reviews of qualitative studies in other topic areas. Searching CINAHL in addition to MEDLINE can add considerably to the resources required for systematic reviews, and it is not clear whether this additional effort can be justified in terms of unique studies identified.

When conducting database searches for the Public Health Research Consortium (http://phrc.lshtm.ac.uk/project_2011-2016_002.html) systematic review “People’s experiences of attempting to make changes in multiple risk behaviours, including perceived barriers and facilitators” [9], we used the CINAHL database in addition to Embase, MEDLINE, PsycINFO and Science Citation Index (SCI) databases. The literature searches retrieved a total of 27,920 records before deduplication with a large number (13,209) identified from CINAHL. When we analysed which databases had contributed to the included studies and which of these were unique, we found that (of the 53 potentially included studies) 43 (81.13 %) of the papers were available from Embase (accessed via OVIDSP and including MEDLINE records), 39 (73.58 %) were available from MEDLINE (accessed via OVIDSP), 36 (67.92 %) from CINAHL (accessed via EBSCO) and 26 (49.05 %) were available from either PsycINFO (accessed via OVIDSP) or Science Citation Index (SCI) (accessed via Web of Science). In addition, we identified that there were nine unique studies, i.e., were only available in one of the databases. The databases that produced these were Embase (3), MEDLINE (2), PsycINFO (1) and SCI (3) while CINAHL did not produce any unique studies. We would not have expected Embase to have contributed so many unique papers nor CINAHL to have made no contribution in terms of unique studies.

Following this finding, we decided to undertake a retrospective analysis of a group of systematic reviews of qualitative studies to investigate CINAHL’s potential contribution in more detail. In particular, we wanted to assess

1. What percentage of included studies were available in CINAHL
2. What percentage of the included studies were unique to CINAHL

## Methods

To develop and refine our inclusion/exclusion criteria, we selected a small convenience sample of ten reviews of qualitative studies [9–19] published between 2007 and 2012 from the Database of Abstracts of Reviews of Effects (DARE). We also used this test set to ensure that there was consistency between the four information specialists carrying out the assessment and data extraction.

We used DARE as it is an excellent source of systematic reviews; it is a database containing critical abstracts of systematic reviews of the effects of health and social-care interventions including prevention, diagnosis, prognosis, treatment and the organisation or delivery of health care. Reviews that cover the wider determinants of health, such as housing, transport and social care are also included.

Potential systematic reviews for DARE are identified by extensive searching including handsearching key journals, scanning websites and running weekly searches of the MEDLINE, PubMed, Embase, PsycINFO and CINAHL databases. The full search strategies used to identify reviews to populate DARE are available at http://www.crd.york.ac.uk/CRDWeb/AboutPage.asp. It was produced by the Centre for Reviews and Dissemination (CRD), part of the National Institute for Health Research (NIHR) and a department of the University of York. The search strategy used to identify potential reviews includes broad search terms such as *review.pt., review.ti.*, *realist review*, *realistic synthesis* and *integrative review* to capture any kind of systematic review as well as terms specifically associated with reviews of qualitative studies such as *meta-synthesis*.

**Fig. 1:**
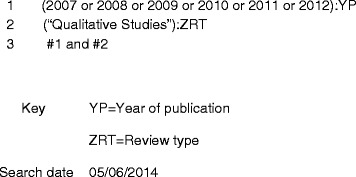
DARE search strategy

We conducted a search of the DARE production database to identify potential reviews for inclusion in our analysis. When reviews are being considered for inclusion in DARE, they are also tagged according to type, so we were able to make use of the “qualitative review” tag in our search strategy (see Fig. 1 DARE search strategy).

Using the sample of reviews from DARE, we developed the inclusion criteria as listed in (see Additional file 1: Table S1 Inclusion criteria), and we proposed that each review had to meet each of the inclusion criteria before being data extracted.

**Fig. 2:**
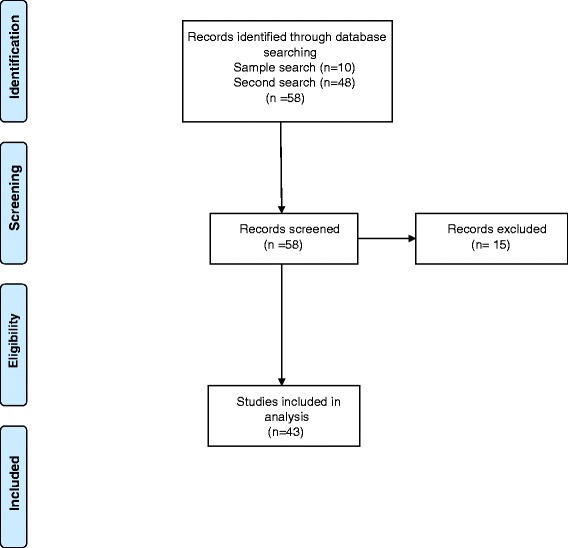
PRISMA 2009 Flow Diagram for the selection of literature

After agreeing the seven inclusion criteria, a further 48 qualitative reviews were then identified from DARE and assessed. At this stage, 15 [20–34] were excluded as they failed one or more of the criteria, leaving us with 33 reviews [11, 35–47, 48–66] eligible for data extraction. We later combined these results with those from the ten reviews initially used in the feasibility study so that we had a total of 43 reviews for analysis as shown in Fig. 2, PRISMA 2009 Flow Diagram, for the selection of literature.

For each review, we extracted the following: review details, publication year, number of included studies, how many databases searched, percentage of potentially included studies available from CINAHL, percentage of unique studies available from CINAHL and whether any other sources were used to identify studies.

For the purposes of this investigation, we recorded the current availability of each of the included studies in each of the databases and, due to logistical constraints, did not attempt to identify whether the database record was available at the time of the original searches or whether it had been identified by the searches. As we were relying upon the description of the searches included in the published paper, there were only a few instances where the original search strategies and search dates were provided in full.

To identify whether or not records were unique to CINAHL, we firstly carried out a check to ascertain whether it was available in CINAHL; secondly, we checked whether it was available in any of the other databases that had been used in the original review. During this second-stage checking, we did identify a number of subscription-only databases that we did not have access to such as CAB Abstracts, SocIndex, Ageline, Health Source Nursing, Academic Search Complete, ProQuest (Dissertations and Theses), PsycARTICLES, Current Contents Connect, Journals@OVID, Sociofile, OTseeker, OT database, Dissertation Abstracts and MIDIRS database. There was also a smaller number of resources that we were unable to use as they were no longer available e.g., Intute, System for Information on Grey Literature in Europe (SIGLE), National Research Register (NRR) or those we could not clearly identify e.g., Digital Dissertations, Conference Proceedings, EBSCO. In these instances, we recorded “not available” but only excluded the review if the “not available” databases constituted more than 50 % of the databases used.

## Results

The 43 reviews included in the analysis at this stage were published between 2007 and 2012 inclusive of the majority (86.1 %) having a publication date of 2011 or 2012. They covered a wide range of topics; there was a small group of seven reviews about pregnancy and childbirth and another smaller group of four reviews exploring older peoples’ experiences of health and care. One review investigated nurses’ experiences of providing services in a primary care setting. Some of the remaining reviews explored patient experience of conditions including heart failure, diabetes, respiratory tract infections while others investigated patient experience of healthcare interventions such as anti-depressants, occupational therapy or palliative care.

Only 6 [15, 35, 36, 40, 41, 55] (14 %) of the 43 reviews did not use any supplementary search methods to identify potential studies for inclusion. The other 37 (86.0 %) reviews all reported some kind of searching activities in addition to using bibliographic databases. Reference checking/footnote chasing was the most frequently mentioned activity (20 reports) (46.5 %) followed by citation searching (described in a variety of ways including forward citation chasing, ancestry searching, citation tracking and backchaining) (10 reports) (23.3 %) and then handsearching key journals and contacting authors (both 8 reports) (18.6 %). Other methods mentioned were scanning conference proceedings, contacting professional bodies, searching for grey literature and looking at the included studies of earlier reviews. Several reviews utilised more than one supplementary search technique.

The number of databases searched per review ranged from 3 to 20, with 16 (37.2 %) of the included reviews searching between 3 and 5 databases, 12 (27.9 %) searching between 6 to 8 databases, 6 (14.0 %) reviews searching 9 to 11 databases and another 6 (14.0 %) reviews searching 12 to 14 databases, while 3 (7 %) reviews had searched over 16 databases.

### Included studies available from CINAHL

**Fig. 3:**
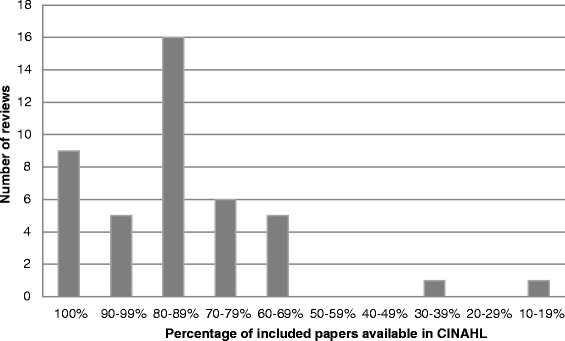
Percentage of papers available in CINAHL

For all 43 reviews, we found that some of the included studies were available in CINAHL (see Fig. 3, Percentage of papers available in CINAHL). For nine (20.9 %) of the reviews, all the studies that had been included in the final synthesis were available in the CINAHL database, so using a robust search strategy, it could have been possible to identify all of these using just this one database. For a further 21 (48.8 %) reviews, there was also a high availability rate of over 80 %. Six (14.0 %) reviews could have retrieved 70.0 to 80.0 % of their included studies from CINAHL while another five (11.6 %) reviews could have retrieved 60.0 to 70.0 % of the included studies. There were two (4.7 %) reviews, however, where the percentage of the included studies available in CINAHL was much lower at 38.5 % and 14.3 %. Both of these reviews [61, 62], written by the same authors, were about the experience of being a kidney donor so not substantially different in topic from the majority of the reviews where CINAHL had appeared to be a good source of primary studies.

### Unique studies available from CINAHL

For 18 (41.9 %) out of 43 of the reviews included in our sample, the CINAHL database had contributed at least one unique study (see Additional file 2: Table S2, Potential unique included studies available from CINAHL) and, for 5 (11.6 %) of these [19, 37, 40, 53, 64], we found that all the included studies were available in the CINAHL database and had the potential to be retrieved from the database assuming a good strategy and appropriate indexing were in place.

Our analysis found that the percentage of unique studies available from the CINAHL database ranged from 3.3 to 33.0 %. However, 7 (38.9 %) of these 18 reviews, where one or more unique studies was available in CINAHL, had originally searched additional databases that we could not access either because they were no longer available or required a subscription. Consequently, the findings regarding CINAHL’s contribution of “unique” studies can only be based on 11 (61.0 %) of the 18 reviews (see Additional file 3: Table S3, Number of databases not available for searching). The median % of unique studies identified from CINAHL in these 11 reviews was 9.09 %, while the range had a lowest value of 5.0 % and a highest value of 33.0 %.

## Discussion

As we had easy access to the DARE database and its collection of systematic reviews, we decided to carry out a retrospective analysis of a sample of systematic reviews of qualitative studies rather than prospectively identifying reviews that may have been eligible for our analysis. One of the consequences of this choice was that we were reliant upon what was reported in the published papers regarding the searching process such as the databases used, database search strategies, supplementary search techniques applied and whether any of the sources had identified unique studies. As all the search strategies were not given in full, there was not sufficient information to base our analysis upon their performance in terms of sensitivity, precision and number needed to read (NRR), so we had to use the availability of records within CINAHL and the other databases as a measure of performance. This approach cannot take account of search strategy design, database interface and accuracy of database indexing, all factors that can affect the retrieval of relevant records. Ideally a planned future analysis would be based upon prospective data so these factors and their impact can be considered.

86.0% (37 out of 43) reviews had used at least one supplementary search technique (including reference checking, citation searching, handsearching key journals and contacting authors) to identify potential studies while some reviews had used several methods. Review authors reported the additional methods used to identify studies in some detail although few of them reported whether or not this extra effort had led to the identification of further studies not identified by any of the database searches. Gomersall et al. 2011 [44] reports that using reference lists of the relevant literature as well as database searching led to “the identification of 38 relevant articles” although it is not clear how many, if any, of the included studies were identified uniquely from the searches of reference lists. Malpass et al. 2012 [67] used both checking reference lists and contacting authors in addition to database searching and reports that “This brought to light one relevant paper which, after critical appraisal, was included in the synthesis”. Steen et al. 2012 [59] states that “backchaining of these papers identified a further six of potential relevance” but, again, it is not clear whether any of these six papers was uniquely identified in this way or whether it was subsequently included in the review. A retrospective analysis of the performance of one or more databases as a source of potential studies for a systematic review requires more detailed reporting than is presently available in published reports. The selection of databases to use for future reviews could be informed by past performance if information about the source of studies was reported more fully.

Although our retrospective analysis showed that for five reviews, all their included studies were available in the CINAHL database, using this information to prospectively select which databases to use in future reviews has limitations. It assumes that past performance could be a predictor of future performance whereas this is unknown. Changes in both thesaurus terms and journal coverage, for example, could have an impact upon database performance.

The 43 reviews included in our sample covered a wide range of topic areas with few distinguishable groups. We did consider whether topic could have had any impact upon the likelihood of CINAHL’s ability to contribute unique studies but, looking at the 11 reviews where searches of CINAHL could have identified studies not available in other databases, no clear topics or themes emerge. The small group of reviews investigates topics covering an equally wide range of topic areas as examined by the overall sample as follows: experiences of breastfeeding, old people’s experience of dependency, fathers’ experience of birth, patient experience of being in isolation, preoperative communication and occupational therapy home visits.

While identifying the evidence for reviews of quantitative evidence synthesis, emphasises the importance of finding all the available studies [1–3]; searching for evidence in reviews of qualitative evidence can have a different aim. Booth et al. [68] describes this as “Their intention is not to identify all literature on a particular topic. While the aim is to identify specific groups of papers that possess characteristics that are relevant to the phenomenon being studied, this need not imply statistical representativeness”. Consequently, being able to retrieve unique papers may be of less significance so long as sufficient papers have been identified to provide sufficient coverage of broad themes and representative viewpoints.

### Limitations

As our study was a retrospective audit of the search methods of a sample of systematic reviews of qualitative studies undertaken by other researchers, we did not have access to the original search results and search strategies. Consequently, we were unable to evaluate the performance of the original search strategies in terms of sensitivity and precision; our study focuses solely upon the current availability of the included studies in the CINAHL database as a performance measure.

The sample of reviews that we used to assess the value of searching CINAHL had all originally used this database as a source of potential-included studies. We did not investigate the reviews that had not used CINAHL as one of the databases for their search. Extending the analysis to include this additional group of studies could be valuable, if it were possible to identify a sufficiently large group.

The lack of access to a number of the databases that had been used by the original reviews limits how confident we can be about whether the CINAHL database can identify unique studies for systematic reviews of qualitative studies in topic areas beyond nursing. This limitation is likely to be met by others attempting to carry out a retrospective study of this kind, especially as electronic resources are discontinued and database subscriptions withdrawn. Nevertheless, using the smaller sample of reviews not affected by this restriction, we were still able to identify 11 reviews where unique studies had been identified by using CINAHL.

## Conclusions

All 43 reviews of qualitative studies in our sample had included studies that were available in CINAHL. In addition, for 21 % (9/43) of the reviews, all the included studies were available in CINAHL. So, assuming a rigorous search strategy and accurate indexing, we could expect CINAHL to be a good source of primary studies for qualitative evidence syntheses.

We also found some indication that CINAHL had the potential to provide unique studies for systematic reviews. Eleven reviews, where we were able to recheck all the databases used by the original review authors, had included a study that was uniquely identified from the CINAHL database.

## Additional files

Additional file 1: Table S1. Inclusion criteria. (DOCX 13 kb)
Additional file 2: Table S2. Potential-included studies available from CINAHL. (DOCX 14 kb)
Additional file 3:Table S3. Number of databases not available for searching. (DOCX 17 kb)
