# Erratum to: What value is the CINAHL database when searching for systematic reviews of qualitative studies?

**DOI:** 10.1186/s13643-015-0128-x

**Published:** 2015-11-20

**Authors:** Kath Wright, Su Golder, Kate Lewis-Light

**Affiliations:** Centre for Reviews & Dissemination, University of York, York, UK; Department of Health Sciences, University of York, York, UK

## Erratum

After publication of [1] it came to the authors’ attention that three percentage (%) symbols were missed upon publication of their manuscript. The incorrect statement present in the Abstract and Results is “The median number of unique studies was 9.09; while the range had a lowest value of 5.0 to the highest value of 33.0”. The correct statement is “The median % of unique studies was 9.09%; while the range had a lowest value of 5.0% to the highest value of 33.0%”. This has been updated in the original article.
