# Supplementary material for: What value is the CINAHL database when searching for systematic reviews of qualitative studies?
Source: Syst Rev. 2015 Jun 26;4:104. doi: 10.1186/s13643-015-0069-4 (PMC4532258; doi:10.1186/s13643-015-0069-4)
Supplement: Additional file 1: Table S1. — Inclusion criteria. (DOCX 13 kb) [file 13643_2015_69_MOESM1_ESM.docx]

**Table S1 Inclusion criteria**

| Criteria 1  Does the review contain purely qualitative studies? (*We did not include any reviews that used a mixed methods approach i.e. included both qualitative and quantitative studies in their synthesis)* |
| --- |
| Criteria 2  Was it published between 2007 and 2012 inclusive? |
| Criteria 3  Is there a list of all the databases searched? |
| Criteria 4  Is CINAHL one of the databases searched? |
| Criteria 5  Are all the included studies listed? (*Either in a table or from the article’s reference list)* |
| Criteria 6  Are at least half of the databases accessible to us via University of York subscriptions, freely available via the Internet or via colleagues? |
| Criteria 7  Is the review published in the English language? |
