# Supplementary material for: What value is the CINAHL database when searching for systematic reviews of qualitative studies?
Source: Syst Rev. 2015 Jun 26;4:104. doi: 10.1186/s13643-015-0069-4 (PMC4532258; doi:10.1186/s13643-015-0069-4)
Supplement: Additional file 2: Table S2. — Potential-included studies available from CINAHL. (DOCX 14 kb) [file 13643_2015_69_MOESM2_ESM.docx]

**Table S2 Potential unique included studies available from CINAHL**

|  | **How many databases searched?** | **% Unique study/ies available from CINAHL?** |
| --- | --- | --- |
| Schmied 2011 | 14 | 3.26%  (1 /31) |
| Abad 2012 | 9 | 5%  (1/20) |
| Lawrence 2012 | 8** | 5.13%  (2/39) |
| Gomersall 2012 | 6** | 5.26%  (2/38) |
| Neubeck 2012 | 3 | 5.88%  (2/34) |
| Munn 2011 | 14** | 6.66%  (1/15) |
| Malpass 2012 | 6 | 7.14%  (1/14) |
| Steen 2012 | 6 | 8.69%  (2/23) |
| Palacios-Cena 2011 | 4 | 9.09%  (2/22) |
| Gill 2012 | 10** | 10%  (1/10) |
| Vottero 2012 | 13 | 12.5%  (1/8) |
| Whalley Hammell 2007 | 3 | 12.5%  (1/8) |
| Mason 2012 | 4 | 15.38%  (2/13) |
| Scope 2012 | 16** | 16.66%  (1/6) |
| Chan 2012 | 6 | 18.18%  (2/11) |
| Bradshaw 2012 | 6 | 19.35%  (6/31) |
| Nolan 2009 | 3** | 30.77%  (4/13) |
| Atwal 2011 | 7 | 33%  (1/3) |

** indicates that we were not able to search all the original databases to ascertain

whether studies were unique to CINAHL
