# Supplementary material for: What value is the CINAHL database when searching for systematic reviews of qualitative studies?
Source: Syst Rev. 2015 Jun 26;4:104. doi: 10.1186/s13643-015-0069-4 (PMC4532258; doi:10.1186/s13643-015-0069-4)
Supplement: Additional file 3:Table S3. — Number of databases not available for searching. (DOCX 17 kb) [file 13643_2015_69_MOESM3_ESM.docx]

**Table S3 Number of databases not available for searching**

| **Review details** | **How many databases searched by original review?** | **How many databases not available** | **Which databases not available** |
| --- | --- | --- | --- |
| Abad 2012 | 9 | 0 | N/A |
| Agudelo-Suarez 2012 | 14 | 0 | N/A |
| Atwal 2011 | 7 | 0 | N/A |
| Bradley 2011 | 7 | 0 | N/A |
| Bradshaw 2012 | 6 | 0 | N/A |
| Chan 2012 | 6 | 0 | N/A |
| Child 2012 | 8 | 0 | N/A |
| Clark 2012b | 10 | 0 | N/A |
| Gill 2012 | 10 | 3 | Academic Search Complete, Proquest Dissertation and Theses, PsycArticles |
| Gomersall 2012 | 6 | 1 | CAB Abstracts |
| Lawrence 2011 | 8 | 0 | N/A |
| Lorenc 2012 | 20 | 4 | AEGIS, BL Direct, Current Contents Connect  NRR |
| Lundgren 2012 | 5 | 1 | Journals OVID |
| Mahant 2011 | 5 |  | Sociofile |
| Malpass 2009 | 5 | 0 | N/A |
| Malpass 2012 | 6 | 0 | N/A |
| Mason 2012 | 4 | 0 | N/A |
| Monforte-Royo 2012 | 6 | 0 | N/A |
| Morgan 2012 | 11 | 0 | N/A |
| Munn 2011 | 14 | 5 | Current Contents, Dissertation Abstracts, Intute, Mednar, Sociological Abstracts |
| Nagata 2012 | 4 | 0 | N/A |
| Neubeck 2012 | 3 | 0 | N/A |
| Nolan 2009 | 3 | 1 | MIDIRS |
| Palacios-Cena 2011 | 4 | 0 | N/A |
| Peoples 2011 | 4 | 0 | N/A |
| Perry 2011 | 9 | 0 | N/A |
| Robinson 2008 | 12 | 2 | National Research Register, SIGLE |
| Schmied 2011 | 14 | 4 | Current Contents, Meditext, Nursing Consult, MIDIRS |
| Scope 2012 | 16 | 1 | HEED |
| Shilling 2012 | 13 | 0 | N/A |
| Smithson 2010 | 18 | 0 | N/A |
| Smithson 2011 | 5 | 0 | N/A |
| Smithson 2012 | 7 | 0 | N/A |
| Steen 2012 | 6 | 0 | N/A |
| Tan 2012 | 9 | 1 | Psyc Articles |
| Taylor 2011 | 5 | 0 | N/A |
| Tong 2012 a | 4 | 0 | N/A |
| Tong 2012 b | 4 | 0 | N/A |
| Tonkin-Crine 2011 | 5 | 0 | N/A |
| Vottero 2012 | 13 | 7 | Current Contents, EBSCO Host Health Source: Nursing/Academic edition; Psychologic and Behavioural Sciences collection, Nursescribe, Proquest Dissertations & Theses, Psychlit, Sociological Abstracts |
| Walsh 2012 | 8 | 0 | N/A |
| Whalley Hammell 2007 | 3 | 0 | N/A |
| Zayac 2009 | 3 | 0 | N/A |
